# Strawberry Fungal Leaf Scorch Disease Identification in Real-Time Strawberry Field Using Deep Learning Architectures

**DOI:** 10.3390/plants10122643

**Published:** 2021-12-01

**Authors:** Irfan Abbas, Jizhan Liu, Muhammad Amin, Aqil Tariq, Mazhar Hussain Tunio

**Affiliations:** 1 School of Agricultural Equipment Engineering, Jiangsu University, Zhenjiang 212013, China; dr.iabbas@yahoo.com (I.A.); mazharhussaintunio@sau.edu.pk (M.H.T.); 2 Institute of Geo-Information & Earth Observation, PMAS Arid Agriculture University, Rawalpindi 46300, Pakistan; m.amin@uaar.edu.pk; 3 State Key Laboratory of Information Engineering in Surveying, Mapping and Remote Sensing (LIESMARS), Wuhan University, Wuhan 430079, China; aqiltariq@whu.edu.cn

**Keywords:** disease classification, convolutional neural network, deep learning, precision farming, Leaf scorch disease, EfficientNet

## Abstract

Plant health is the basis of agricultural development. Plant diseases are a major factor for crop losses in agriculture. Plant diseases are difficult to diagnose correctly, and the manual disease diagnosis process is time consuming. For this reason, it is highly desirable to automatically identify the diseases in strawberry plants to prevent loss of crop quality. Deep learning (DL) has recently gained popularity in image classification and identification due to its high accuracy and fast learning. In this research, deep learning models were used to identify the leaf scorch disease in strawberry plants. Four convolutional neural networks (SqueezeNet, EfficientNet-B3, VGG-16 and AlexNet) CNN models were trained and tested for the classification of healthy and leaf scorch disease infected plants. The performance accuracy of EfficientNet-B3 and VGG-16 was higher for the initial and severe stage of leaf scorch disease identification as compared to AlexNet and SqueezeNet. It was also observed that the severe disease (leaf scorch) stage was correctly classified more often than the initial stage of the disease. All the trained CNN models were integrated with a machine vision system for real-time image acquisition under two different lighting situations (natural and controlled) and identification of leaf scorch disease in strawberry plants. The field experiment results with controlled lightening arrangements, showed that the model EfficientNet-B3 achieved the highest classification accuracy, with 0.80 and 0.86 for initial and severe disease stages, respectively, in real-time. AlexNet achieved slightly lower validation accuracy (0.72, 0.79) in comparison with VGGNet and EfficientNet-B3. Experimental results stated that trained CNN models could be used in conjunction with variable rate agrochemical spraying systems, which will help farmers to reduce agrochemical use, crop input costs and environmental contamination.

## 1. Introduction

Plant diseases lead to the loss of modern farming production. The emergence of plant diseases has a negative impact on agricultural crop yield. Plant diseases are not only a threat to global food security, but they can also have terrible impacts for small farmers whose incomes depend on crop production. Therefore, if plant infections are not identified at early stages, they will decrease crop yield and food shortage will arise [1]. A healthy plant must be protected from disease to ensure the quality and quantity of crop yield, as they are harshly affected by diseases [2,3].

There are several diseases that affect crop production and also cause economic, social, and environmental problems. Strawberries are one of the most sensitive and important crops in the world. Strawberries have high nutritional content and commercial value, and are a major fruit for daily consumption [4,5]. Strawberries are easily infected by several phytopathogenic fungi, bacteria, and viruses [6,7,8]. Strawberry leaf scorch (Diplocarpon earlianum) is one of the most common and a severe leaf diseases of the strawberry crop, caused by a species of fungus. The marks of leaf scorch disease consist of many small irregular purple spots that appear on the outward leaf’s surface. It has a similar disease cycle to other fungal diseases and it is managed in the same way. These diseases can cause significant economic damage. Control of strawberry leaf scorch disease is important because it is responsible for losses ranging from negligible to severe in strawberries. Leaf scorch disease affects the yield of the strawberry crop. The application of appropriate fungicides at the early stage of leaf scorch disease can reduce crop production losses. Plant disease and pest control at the early stages of infections is essential for higher agricultural production [9].

It also reported that more than 50% of crop production losses are the result of different diseases and pest outbreaks [10]. Several efforts have been made to avoid crop losses due to these diseases. In agriculture, expensive approaches and numerous agrochemicals are used to control these diseases and pests. The extensive usage of these agrochemicals approaches is harmful to plant and human health. Furthermore, these agrochemicals also increase crop input costs [11]. Unnecessary application of agrochemicals can lead to ecological deprivation, for example, the erosion of useful soil components or the addition of poisonous ingredients in the soil [12]. For this reason, precise and quick recognition of plant diseases is important in contemporary farming [13]. In the initial stages, the identification of plant disease can serve as the key role for crop prevention. Indeed, it will enhance the quantity and quality of agricultural products. It is important to note that early follow-up is important for the proper selection of disease management techniques and to halt the spread of the infection in healthy plants [14]. In general, recognition and classification of diseases are noted by simple visual observation. However, this procedure must be regularly checked by professionals [15] because it is also possible that farmers can wrongly diagnose the diseases because of limited knowledge.

The traditional method of checking plant disease infections is done by hiring professional pathologists who can diagnose the disease correctly. However, this traditional method needs more manual labor and time, and the spread of the disease in the field cannot be accurately predicted [16]. Moreover, these difficulties are combined with the reduction in workers in the farming region. Visual observations by skilled authorities remain a key method for identifying plant diseases in rural regions of developing nations. Visual observations involve constant observing by specialists, which can be costly in big farms [17,18]. Furthermore, in several isolated regions, farmers may have to travel long distances for hiring specialists, which is economically very expensive and time-consuming for small farmers. However, this method can only be implemented in limited regions, and cannot be prolonged in a positive way. The high cost and low proficiency of manual disease identification deter the fast progress of existing farming [19]. Therefore, automatic and accurate disease identification systems are crucial for agriculture production.

Autonomous farming technologies are being implemented to control plant diseases. The use of agrochemicals and other expensive approaches can be shortened by using intelligent systems. In precision farming technologies, many intelligent sensor systems and automation methods are used. For intelligent farming, such as agrochemical spraying on only the relevant region, it is necessary to identify the specific disease target area in the field. Mobile operators, stationary stations, sensor systems and machines are used for precision sensing in agriculture. Initially, precision farming was used to distribute fertilizers according to diverse soil situations. Later, precision agriculture advanced for automatic control of agricultural machines and implements, intelligent machines and practices, on-farm investigation and mechanized controlling of farming production structures [20]. Data collection by sensors installed on machinery is non-destructive and can be used at a large scale. Soil and aerial images are frequently important methods in precision agriculture [21], and are used for the identification and classification of diseases. Intelligent classification of plant diseases is an important investigation method, as it can demonstrate the assistance of observing huge crop areas to identify disease symptoms by observing the plant leaves [22,23,24]. Therefore, looking for a faster, more automatic, cheaper and more precise process of identifying plant diseases is important.

Progress in artificial intelligence expertise has given the approach for the improvement of mechanized systems capable of achieving quicker and more precise outcomes in detecting plant diseases. Today, artificial intelligence-based systems are widely used to automatically identify various diseases [25]. Over the past decade, several outdated machine learning simulations have been planned for identifying and categorizing plant infections. Machine vision and deep learning methods for disease recognition have been extensively investigated over the past twenty years [26]. Image processing has been used extensively in farming, such as that used for weed identification, fruit classification [27], also image processing used to detect and categorize plant diseases [28] and classifying of different disease signs [29]. In recent times, many researchers have used deep learning for recognition and studied various plant leaves diseases in depth [30]. Image-based methods are fast and precise in identifying plant infections [31]. Digital images give significant data elements that can be examined to create basic information for the number of uses. Machine learning (ML) can be used to sense pests, parasites, and deficiency of nutrients in plants [32].

In recent years, deep convolution neural networks have been extensively used in the farming sector for example in weed, pest, and disease identification, fruit, flowers and plant classification for yield assessment, and also in autonomous vehicles for navigation purposes [33,34,35]. Deep learning can be classified into three categories according to the technique and process of the research. Deep convoluted neural networks have been used as the most advanced computer vision (CV) in various fields since 2014, and are the most popular for identifying crop diseases because of their huge classification precision detection accuracy of imageries [36]. Deep convolution neural networks (DCNN) have an amazing capability to extract multifaceted structures from pictures [37,38]. Images have been widely used as an influential instrument for classifying and identifying targets [39]. Convolution Neural Network (CNN) is one of the most popular machine learning (ML) methods, according to published reports for the classification of crop diseases. Fujita et al. [40] reported a novel real-world plant disease recognition method that included seven kinds of diseases. They used CNN-based classification systems with an average accuracy of 82.3%. Sladojevic et al. [41] suggested a different method to distinguish 13 diverse plant diseases by deep convolutional neural networks. Another author reported a powerful deep learning-originated device for real-time use that can identify nine various diseases of tomato plants [42].

Some professionals are using the bandwidth of noticeable light that can be taken by cameras at a comparatively low price, and have generally engaged in a particular disease category [43]. Since a single tuber can infect multiple diseases, it will not be sufficient for real-time applications [44]. Ferentinos et al. [45] proposed the CNN technique for plant leaf recognition using the GoogLeNet model. The proposed technique was able to identify damaged leaves with a recognition rate of >94%, even when only 30% of the leaf was damaged. Mohanty et al. [30] used CNN for identifying crop species and diseases based on a public dataset of images using GoogLeNet and AlexNet training models. Based on the color, grayscale, and leaf segmentation, the proposed model was 99.35% accurate. The demonstration of deep learning procedures depends mostly on the quality of the dataset than on other ordinary machine learning methods. Some recent studies stated above show that there is an improvement in the use of deep learning architectures in the identification of leaf diseases in plants. Still, there are gaps that need to be explored in identifying plant leaf diseases, particularly around the use of new deep learning architectures. In particular, there is an inevitable need for effective models that are faster, and have more minor limitations and higher classification accuracies.

This study compares the performance of four CNNs models (EfficientNet-B3, VGG-16, AlexNet and SqueezeNet) for the identification and classification of the initial and severe stage of leaf scorch disease in the strawberry crop. The study also evaluates the effect of initial and severe stage of leaf scorch disease on the performance of CNN models. This article introduces a new approach for real-time disease identification in the strawberry field. For this purpose, the trained CNNs models (EfficientNet-B3, VGG-16, AlexNet and SqueezeNet) integrated with a machine vision system for real-time image acquisition and identification of leaf scorch disease in the strawberry field. Furthermore, the performance of the trained CNNs models was evaluated under natural sunlight and artificial lighting arrangements for image acquisition and better identification of leaf scorch disease in the strawberry field.

## 2. Materials and Method

### 2.1. Convolutional Neural Network Models

The convolutional neural network is a multilayer deep learning network designed to process data, including image, audio, and video. In CNN, the image is obtained from the input terminal, and the image features are filtered out to the pooling layer through the convolution layer to sort out the new image features. The convolutional layers comprise a filter and a trigger function, and the leaf images will be classified into healthy or infected leaves using convolutional neural networks based on the extracted characteristics.

**Figure 1:**
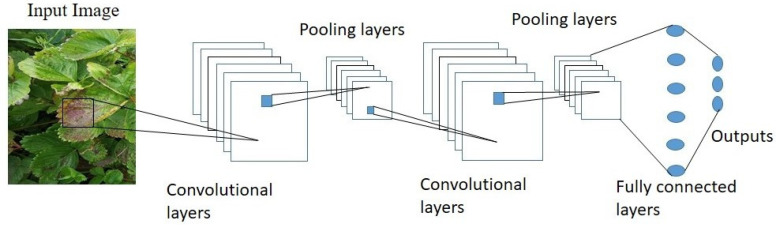
Convolutional neural network for the classification of strawberry diseases.

For identifying plant types and their diseases, the CNN can demonstrate better classification accuracy than other typical characteristics extraction approaches [46]. A traditional CNN architecture contains primarily convolution layers, grouping layers, full contact layers, and output layers [47], as shown in Figure 1. There are various DCNN models with their own features that are suggested each year. We focus on the two most popular architectures, namely AlexNet [48], VGG16 [49], and a smaller faster architecture SqueezeNet [50], and the performance of the proposed models compared with the newly introduced architecture EfficientNet [51]. These most precise architectures have been trained for the classification of the initial and severe stages of leaf scorch disease in strawberry plants. All four CNN models, namely, AlexNet, VGG, SqueezeNet and EfficientNet, were trained and tested for the identification of leaf scorch disease in strawberry plants. EfficientNet is the most recent devolved model.

#### 2.1.1. AlexNet

AlexNet was proposed by Alex Krizhevsky et al. [48]. This model was presented for the first time in the ImageNet Large Scale Visual Recognition Challenge (ILSVRC-2012) in 2012 as part of the Image Classification Task Competition. Furthermore, the model won by a large margin. AlexNet turns into the initial step for CNN’s new trend. The AlexNet architecture carries the size of the input image to 227 × 227. It has 60 million parameters and 650,000 neurons. AlexNet has five convolutional layers and three interconnected layers. First, the two convolutional layers are normalized, and the third and fourth are followed by a layer of maximum accumulation; they are directly connected, and a max-pooling is followed by layers of the fifth convolutional layer. The last layer (FC8) has a class possibility of the coming input image. These possibilities are categorized from the softmax classifier. One of the innovatory facts of AlexNet was its fast training by GPU. Multicore GPUs have increased the speed of learning on very large image data.

#### 2.1.2. VGG-16

The VGGNet [49] convolutional neural network architecture was introduced by some scholars of the visual geometry group from the University of Oxford and (DeepMind) Google. VGGNet won the runner-up position in the Image Classification Contest held in 2014. The model achieved an error rate of 7.5% in the top five in the rating set, a result that earned it second position in the competition. VGG-16 inherited the AlexNet (2012) architecture design ideas and went from eight to 16 layers on the AlexNet architecture. VGG-16 consists of 13 convolution layers in which each two convolution layers have a pooling layer at the end and three fully connected layers. The VGG-16 follows this arrangement of two convolution layers having a pooling layer during the whole architecture. It always uses 3 × 3 filters for Convolution. It takes an image input size of 224 × 224 pixels and the model has approximately 138 million parameters.

#### 2.1.3. SqueezeNet

The SqueezeNet model was proposed by Iandola et al. [50] in 2016. SqueezeNet is a smaller and faster architecture with fewer parameters and better classification performance and is also suitable for integration with smaller computing devices such as single-board computers or mobile phones. SqueezeNet has eight fire modules, each consisting of a compression layer with 1 × 1 filters (downward input channel of 3 × 3 filters) and an extension layer with a combination of 1 × 1 and 3 × 3 filters. By doing so, the number of incoming connections into 3 × 3 filters is reduced. The architecture begins with a convolutional layer, followed by eight fire modules, followed by a convolutional layer, and a softmax classifier. It requires an image input size of 227 × 227 pixels and the model has around 1.2 million parameters.

#### 2.1.4. EfficientNet

The EfficientNet model was proposed by the Google research brain team in 2019 at the international conference on machine learning [51]. In the ImageNet classification methods, the EfficientNet model is one of the latest models with a parameter of 66M and an accuracy of 84.4%, also known as a group of CNN models. EfficientNet has eight models from B0 to B8, and if we increase the number of models, the remarkable accuracy of the model will also increase, but the parameters will not increase considerably. The latest version of EfficientNet is EfficientNet-B7, and it has the highest accuracy with fewer parameters. EfficientNet uses the mobile inverted bottleneck convolution (MBConv). The MBConv block receives two inputs, the first one is data and the second is arguments of the block. A set of attributes, such as input filters, output filters, expansion rate, and compression rate, are used in an MBConv block. EfficientNet also performs better for complex data. EfficientNet-B3 takes the input images of 300 × 300 pixels and architecture with approximately 12 million parameters.

### 2.2. Dataset Description

**Figure 2:**
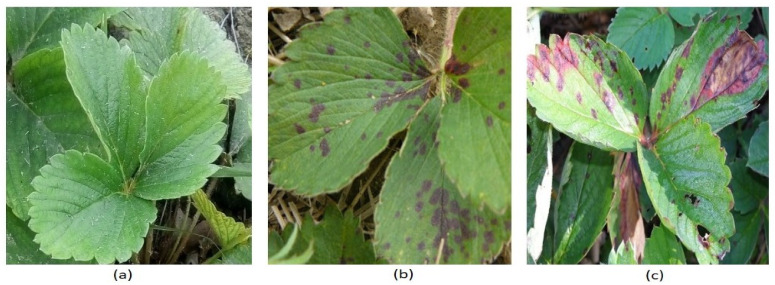
Sample of strawberry leaf images from dataset. (**a**) Healthy (**b**) Initial disease stage of leaf scorch and (**c**) Severe disease stage of leaf scorch.

Images of strawberry leaves (healthy and infected) were taken from the strawberry fields in southern Punjab (31°21′41.99″ N, 70°58′10.99″ E), Pakistan, during the 2020 crop season. A digital camera (Canon PowerShot SX530 Digital Camera) captured colour images, at a ratio of 16:9, with a resolution of 1920 × 1080 pixels, of diseased (leaf scorch) and healthy strawberry leaves (Figure 2). These images were taken at morning, noon, afternoon and evening, under different light intensities, environments and from varying positions to encompass all possible situation for training the CNNs models. Images were captured based on visual symptoms to categorize the initial and severe stages of the disease in strawberry plants. Identification of initial symptoms of leaf scorch in strawberry plants would be beneficial for more timely diagnosis of diseases so that they can be controlled on time by applying fungicides.

A total of 1689 leaf images were taken, in which 552 were of healthy leaves, 580 were of the initial stage, and 557 were of the severe stage of leaf scorch disease infected leaves. The data augmentation method in deep learning is important for generalizing the models to increases their accuracy, so that the models can be more efficient and functional in real-world field conditions [52]. The augmentation method can help the model to increase the precision of its predictions by training the models from diverse dataset perceptions. Therefore, to improve the robustness of the architecture and increase the number of observations, the data augmentation method was implemented with all datasets [53]. For data augmentation, the rotation technique was performed considering the different shapes and directions of the leaves in the field.

In data augmentation, all images in the dataset were rotated clockwise by 45° degrees (starting from 0° to 315° of the original image). In this way seven new images were achieved from every single image. The total 1689 image dataset was therefore increased by eight times to 13,512 new observations, in which 580 initial stage and 557 severe stage disease images were increased to 4640 and 4456 images, respectively, and 552 healthy leaves images increase to 4416 images.

### 2.3. CNN Models Training and Testing

A dataset containing 13,512 photographs of healthy and diseased (leaf scorch) leaves of strawberry plants was used for the training and testing of CNN models. All the images in the dataset were randomly divided into three splitting ratios of datasets for training, validation and testing of the object-classification CNN models. The splitting ratios of datasets were 80:20 (80% dataset used for training, and 13% for validation and 7% for testing) as shown in Table 1.

For training and testing of the convolutional neural network, all the images used in this study were resized to 224 × 224, 227 × 227, 227 × 227 and 300 × 300 pixels by IrfanView software (Version 5.50, Irfan Skijan, Jajce, Bosnia) for VGG-16, AlexNet, SqueezeNet and EfficientNet-B3 models respectively according to the network input size for more accessible learning, validation and testing processes. The images used for training were not used during testing. All model training and testing processes were performed using a computational processing unit (Nvidia GeForce GTX 1080 Integrated with 8 GB GDDR5X RAM that operates on 256-bit memory) and a Pascal GPU GP104 that operates at a frequency of 1733 MHz) on the operating system Windows 10 64 bits. The keras API running on tensor flow machine learning computational framework was used for model training. The tensor flow framework was developed by Google, and was originally developed to perform large datasets of numerical calculations [54].

To train our convolutional neural network models, we randomly initialized its parameter weights and bias, the parameters are continuously updated through forward propagation and backward propagation during training, and the final ones at the end of the training establish the models. Additionally, several hyper-parameters were selected to obtain the high value of precision. Hyper-parameters directly affect the performance and training speed of neural networks. The hyper-parameter tuning process was used for the optimization of hyper-parameters. The optimal values of hyper-parameters were determined using a trial-and-error process by adjusting their values and selecting the hyper-parameters that give the best results on the validation set. Momentum value was attained as 0.95, batch size 32, the base learning rate was 0.001, and weight decay 0.0005 presented the best results. VGG-16, AlexNet, SqueezeNet and EfficientNet-B3 models were used and trained for diseased and healthy strawberry plant leaf classification using the tensor flow framework. After training, all DCNN models were also tested for the classification of disease-infected leaves and the models were saved.

### 2.4. CNNs Models Evaluation Parameters

The performance of the CNN models was evaluated with different evaluation metrics. Precision, recall, F1Score, and test accuracy metrics were used to evaluate the performance of the convolutional neural network models that were used in training. Validation and test outcomes for all CNN models were adapted in matrices of binary confusion, which are true positive (Tp), false positive (Fp), true negative (Tn), and false negative (Fn) [55]. Healthy leaves indicate as negative (N and 0), and diseased, infected leaves mark as positive (P and 1). The true positive indicates the number of leaves that are appropriately categorized as disease infected, and the true negative indicates the number of leaves that are correctly categorized as non-infected leaves. False-positive is considered a type 1 error, indicating the number of leaves that have been falsely categorized as infected leaves, while a false negative, which is considered a type 2 error, indicates the number of leaves that have been wrongly categorized as uninfected. By using a confusion matrix, the test accuracy, precision, recall, and F1-score will be calculated.

Precision represents the accuracy of a neural network model in the occurrence of a positive recognition, and is measured by Equation (1):

Precision = Tp/(Tp + Fp)(1)

The sensitivity represents the effectiveness of the neural network in which the target is classified and calculated by Equation (2):

Sensitivity (Recall) = Tp/(Tp + Fn)(2)

Test accuracy is the total observation rate of the correctly predicted observation and measured by Equation (3):

Test accuracy = Tp + Tn/ (Tp + Fp + Fn + Tn) (3)

The F1-score is the average of harmonic mean of precision, recall and calculated by Equation (4):

F1-score = 2 × precision × recall / precision + recall(4)

### 2.5. Performance Evaluation of Trained CNN Models for Real-Time Plant Disease Classification

**Figure 3:**
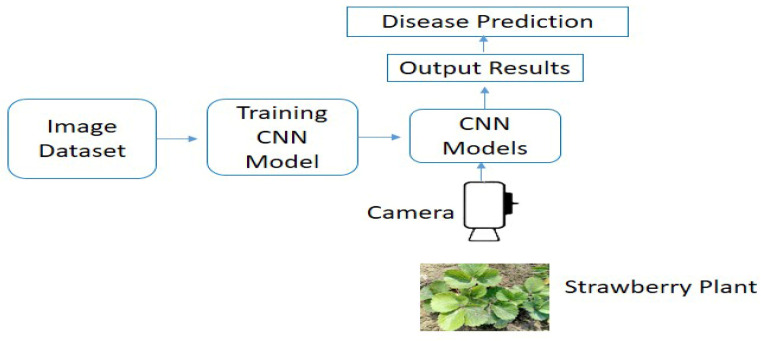
Workflow diagram of CNN models for real time disease identification in field.

A deep learning based unmanned ground vehicle (UGV) was developed for real time plant disease (leaf scorch) recognition in the strawberry field as shown in Figure 3. The vehicle system comprises an electric four-wheeled chassis frame vehicle. The vehicle had 0.60 m of ground clearance and the two wheels spacing was kept at 70 m for moving in strawberry row. The wheel spacing and ground clearance were kept changeable according to the strawberry field conditions. The vehicle system was power-driven by a 24-V lithium battery and automatically drives by remote control (SAGA1-L8B), and the vehicle ground speed is up to 5 km/h. The vehicle was designed to operate within a single strawberry plant row (0.60 m width). The unmanned ground vehicle consisted of a color camera (Logitech C920) for image acquisition in real time and a laptop.

The camera was connected directly to the laptop computer using universal serial bus (USB) cables. Furthermore, the camera acquires 640 × 256 pixels image and covers a 0.50 m × 0.60 m (length × width) area of a single strawberry row. For the identification of disease infected strawberry plants, the trained CNN models were deployed in Intel(R) Core(TM) i7-4712MQX CPU @ 2.30 GHz, Nvidia GTX850M laptop computer. All four trained CNN models (AlexNet, VGG-16, SqueezeNet, and EfficientNet-B3) were implemented in real-time in the strawberry field to identify initial symptoms and severe symptoms of leaf scorch disease in strawberry plants, and the models performance was evaluated by observing the infected plants predictions and by comparing it with manually detected disease plants in the strawberry field (Figure 3).

#### Experimental Plan

**Figure 4:**
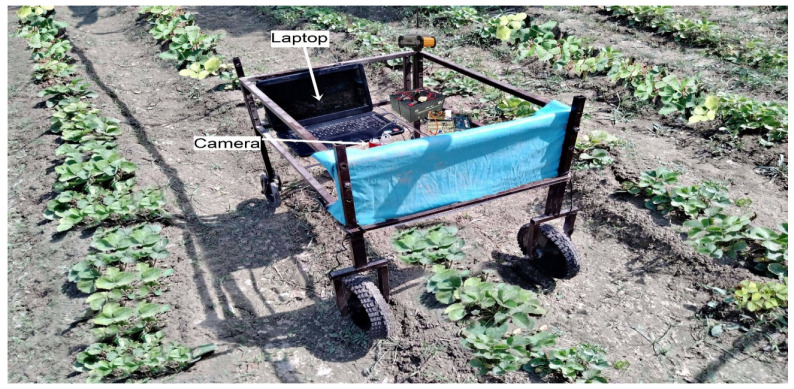
Deep learning based mobile system for real time leaf scorch disease identification in strawberry field.

The field experiment was performed in the strawberry field located in district Layyah (31°21′41.99″ N, 70°58′ 0.99″ E), southern Punjab, Pakistan. The performance of trained CNN models (AlexNet, VGG-16, SqueezeNet, EfficientNet-B3) for the classification of leaf scorch diseased infected plants were evaluated under two different lighting situations (natural and artificial) during the image acquisition in the field experiments (Figure 4). In natural lighting conditions, the natural outdoor sunlight influences the chromatic color variations throughout the image acquisition process. The resulting images show chromatic aberration in the leaves of the strawberry plants that affect the CNN models’ performance. During the natural lighting situation of the field experiment, the wind speed was 2–5 km h^−1^ with an ambient temperature of 27–32 °C and relative humidity of 14–20%.

In the controlled lighting environment for image acquisition, the vehicle system was covered with green cloth so that the imaging sight can be protected from direct sunlight and reduce the chromatic color variations throughout the image acquisition in the strawberry field [56]. In this way the image acquisition quality is enhanced, and CNN models performance improved. Throughout the artificial lighting environment experiment, the wind speed was 2–6 km h^−1^ with the ambient temperature of 25–35 °C and relative humidity of 20–30%. A 50m long single strawberry plant row was selected for real-time field experiments, each experiment was repeated five times and the average classification accuracy values were calculated by evaluation metrics.

## 3. Results

### 3.1. CNNs Models Performance Results

The CNN models (AlexNet, VGG-16, SqueezeNet, EfficientNet-B3) were trained at an 80:20 dataset splitting ratio (80% dataset used for training, 13% for validation, and 7% for testing). The values of recall, F1-score, and precision obtained from all models on the test dataset are provided in Table 2. For all CNNs models, classification accuracy values were recorded in the range of 0.88 to 0.92 and 0.93 to 0.98 for initial and severe disease stages, respectively. The validation results show that the EfficientNet-B3 outperformed than the other CNN models and achieved higher values of accuracy, precision, sensitivity/recall, and F1-score.

The EfficientNet-B3 model achieved the higher values of precision (0.98), recall (0.97), and F1-score (0.97) for severe disease stage as compared to classification values of initial disease stage infected leaves (precision 0.92, recall 0.91, and F1-score 0.91). The EfficientNet-B3 and VGG-16 significantly performed better than AlexNet for the classification of disease leaves (initial stage and severe stage).

The second-best model was VGG-16, which provided higher values of precision (0.96), recall (0.95), and F1-score (0.95) for the severe disease stage, and the classification accuracy values decrease for initial stage classification. The model recorded values of precision, recall and F1-score were 0.91, 0.90 and 0.90, respectively. SqueezeNet was less effective in the classification of the initial disease stage and attained the lowest values of precision (0.87), recall (0.88), and F1-score (0.87). However, for the classification of severe disease stage, SqueezeNet achieved significant classification values of precision (0.93), recall (0.92), and F1-score (0.92). The validation results show that the SqueezeNet model for the classification of fungal leaf disease recorded lower values of precision, recall and F1-score as compared to the other two models.

**Figure 5:**
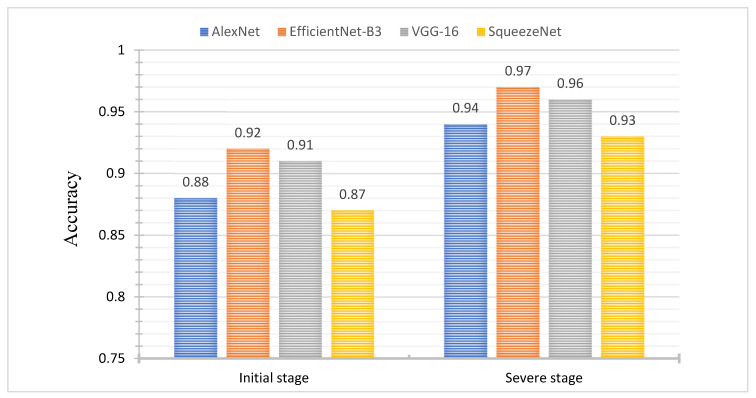
Accuracy of AlexNet, EfficientNet, VGG-16 and SqueezeNet for the classification of initial and severe stage of leaf scorch disease infected leaves.

The test accuracy is also an effective performance metric used to evaluate the performance of the CNN models. The EfficientNet-B3 model achieved higher accuracy with 0.92 and 0.97 for initial and severe disease stage classification as compared to the VGG-16, AlexNet and SqueezeNet models. The VGG-16 model attained slightly lower accuracy with 0.91 and 0.96 for initial and severe disease stages, respectively, on the other hand SqueezeNet attained the lowermost accuracy values with 0.87 and 0.93 for initial and severe disease stages, respectively (Figure 5). AlexNet also achieved lower classification accuracy in comparison with VGG-16 and EfficientNet-B3. The validation results reveal that overall EfficientNet-B3 model performed better than the other three CNNs models (VGG-16, SqueezeNet, AlexNet) for the classification of both disease stages (initial stage, severe stage).

#### CNNs Models Inference Time

The average inference time for all the CNN models is reported in Table 2. The model SqueezeNet recorded that the lowest inference time ranged from 66 to 76 milliseconds as compared to other CNN models. The highest inference time was recorded for the VGG-16 model, which ranged from 349 to 355 milliseconds. There was no significant difference in inference time during initial and severe stage disease infected strawberry leaves. The AlexNet model recorded the second lowest inference time ranging from 212–222 milliseconds.

### 3.2. Performance of CNN Models in Real-Time Field Experiments

#### 3.2.1. Field Experiment with Natural Lighting

The deep learning-based leaf scorch disease classification mobile system was tested in a strawberry field under a natural lightening environment. In natural lighting situations, the direct sunlight was influencing the image acquisition process that causes chromatic color variations and affects the image quality. All four trained CNN models (AlexNet, SqueezeNet, VGG-16, EfficientNet-B3) were implemented in the strawberry fields to identify initial symptoms and severe stages of leaf scorch disease in strawberry plants.

EfficientNet-B3 and VGG-16 performed better than AlexNet and SqueezeNet to correctly identify the infected leaves in real-time during the field experiment (Table 3). The EfficientNet-B3 and VGG-16 models showed significant performances for identifying severe disease stage (leaf scorch) infected leaves and achieved high precision (0.83, 0.80), recall (0.81, 0.78), and F1-score (0.81, 0.78) values. However, for the classification of the initial stage of leaf scorch infected leaves, the values of precision (0.73, 0.70), recall (0.70, 0.67) and F1-score (0.71, 0.68) were considerably lower. SqueezeNet seemed to be unsuccessful at identifying the initial disease stage infected leaves with lower values of precision (0.64), recall (0.61), and F1-score (0.62). However, it showed significant performance for identifying severe disease stage (leaf scorch) infected leaves and achieved better precision (0.73), recall (0.71), and F1-score (0.71) values.

**Figure 6:**
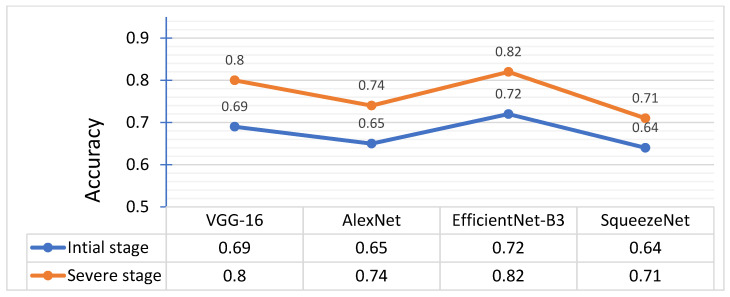
Accuracy of AlexNet, EfficientNet, VGG-16 and SqueezeNet for the classification of initial and severe stage of leaf scorch disease.

The EfficientNet-B3 model also achieved higher classification accuracy (0.82) for the severe disease stage, while for initial disease symptoms, the model accuracy was reduced to 0.72. Similarly, the VGG-16 model achieved a higher accuracy of 0.80 for the classification of severe disease stage as compared to initial disease symptoms, with a 0.69 classification accuracy (Figure 6). On the other hand, SqueezeNet gave the lowest accuracy values with 0.64 and 0.71 for initial and severe disease stages, respectively. EfficientNet-B3 and VGG-16 models performed well for the classification of severe stage leaf scorch infected leaves in strawberry plants during the field experiment.

#### 3.2.2. Field Experiment with Controlled Sunlight Environment

The performance of trained CNN models (AlexNet, VGG-16, SqueezeNet, EfficientNet-B3) was evaluated in a real-world field experiment with artificial lighting arrangements for better image acquisition and classification of diseased infected (leaf scorch) strawberry plants. The disease classification values of precision, recall, and F1 score was higher in the case of EfficientNet-B3 and VGG-16 as compared to AlexNet and SqueezeNet (Table 4). The EfficientNet-B3 and VGG-16 model reported excellent classification values of precision (0.87, 0.85), recall (0.85, 0.83), and F1-score (0.85, 0.83) for severe disease stage (leaf scorch) infected plants. However, the model performance decreased for the classification of the initial disease stage.

Similarly, the AlexNet model achieved higher precision (0.80), recall (0.78), and F1-score (0.78) values for the classification of severe disease symptoms as compared to initial disease symptoms (precision 0.73, recall 0.70 and F1-score 0.71). The SqueezeNet model also achieved lower classification values as compared to other models.

**Figure 7:**
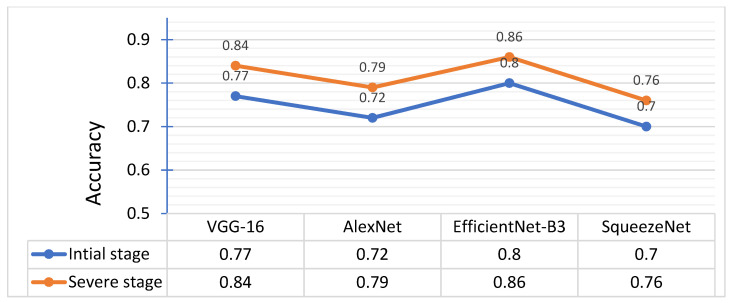
Accuracy of AlexNet, EfficientNet, VGG-16 and SqueezeNet for the classification of leaf scorch disease in strawberry plants.

The EfficientNet-B3 and VGG-16 models also achieved higher accuracy values (0.86, 0.84) for the classification of severe disease, and recorded lower accuracy values (0.80, 0.77) for initial disease symptoms. SqueezeNet presented the lowest accuracy values, with 0.68 and 0.76 for initial and severe disease symptoms, respectively (Figure 7). The highest disease classification accuracy for EfficientNet-B3 makes it an excellent model for real-time applications.

## 4. Discussion

In this study, the applicability of four state-of-the-art CNN models for the classification of diseased (leaf scorch) infected strawberry plants was carried out by using image datasets for training and testing. This research aimed to compare the performance of CNN models by evaluating the accuracy, precision, sensitivity, and F1-Score values in real-time field experiments. A database containing 13,512 photographs of healthy and diseased (leaf scorch) leaves of strawberry plants was used for the training and testing of the CNN models. CNN models present an opportunity for the classification of plant diseases using digital images.

All the image datasets were randomly subdivided into an 80:20 dataset splitting ratio (80% dataset used for training, 13% for validation, and 7% for testing) for training and testing of the object-classification CNNs models. Furthermore, four CNNs models SqueezeNet, VGG-16, AlexNet, and EfficientNet-B3 were trained for the classification of diseased strawberry plant leaves. The performance validation results of the CNN models were calculated with different evaluation metrics, such as sensitivity, accuracy, F-Score, and precision. Table 2 presents the validation results for all four CNN models for the classification of the initial and severe stages of leaf scorch disease infected strawberry leaves.

CNN model validation results show that all the trained models achieved significant values of accuracy, F1-score, precision and recall for the classification of the initial and severe stages of leaf scorch disease infected strawberry leaves. It was also observed that the severe stage of leaf scorch disease infected plants was mostly correctly identified compared to the initial stage of leaf scorch disease. The validation results show that the EfficientNet-B3 achieved the highest classification values of precision (0.98), recall (0.97), and F1-score (0.97) for the classification of the severe stage of leaf scorch disease. Furthermore, for the classification of the initial stage of leaf scorch disease, the highest values of precision (0.92), recall (0.91), and F1-score (0.91) were recorded by EfficientNet-B3. The second-best model was VGG-16, and the model achieved the highest classification values of precision (0.96), recall (0.95), and F1-score (0.95) for the severe stage of leaf scorch disease. Additionally, the model achieved the significant classification values of precision (0.91), recall (0.90), and F1-score (0.90) for the classification of the initial stage of leaf scorch disease. SqueezeNet was found to be less accurate in the classification of both disease stages and achieved low precision, recall and F1-score when compared with other CNN models.

The performance of the CNNs models was also assessed by a test accuracy evaluation metric. According to the performance results reported in Figure 5, the EfficientNet-B3 model attained the highest accuracy among the other CNN models, while the second-best accuracy values were achieved by the VGG-16 model for both stages of leaf scorch disease classification. The EfficientNet-B3 model classification rate is comparable with the most famous ResNet18, ResNet50 models and other version EfficientNet-B4 models that were able to classify disease leaves with an accuracy of 96% [57,58] however, the EfficientNet-B3 model classification rate is higher than the GoogLeNet model classification accuracy values 0.90. Therefore, it can be concluded that the proposed EfficientNet-B3 model is significant for the classification of plant diseases. The average inference time for all the CNN models was also recorded. AlexNet performed better and recorded the lowest inference time, ranging from 109 to 119 milliseconds, compared to other CNN models. The EfficientNet-B3 and VGG-16 models recorded inference time ranging from 212–225 and 344–361 milliseconds, respectively. It also observed no significant difference in inference time during initial and severe stage disease (Table 2).

The proposed models can be integrated with handy devices with minimal computational resources, such as the raspberry pi device or mobile phone. Several researchers also presented real-time object identification with the deep neural network model. Zainab et al. [59] trained tiny yolov2 models implemented in an android mobile phone for real-time work with minimal computational resources. The proposed model achieved 66.3 mean average precision. Ramcharan et al. [60] developed a mobile-based deep learning model for real-time disease identification. A Single Shot Multibox (SSD) model with the MobileNet detector and classifier was used. The model achieved 0.70 accuracy for real-time disease identification. Another researcher developed a Single Shot Multibox Detector for real-time identification, the model was able to identify nine different tomato diseases and pests [43]. All four trained CNN models (SqueezeNet, AlexNet, VGG-16, and EfficientNet-B3) were applied in the strawberry field for the classification of the initial and severe stage of leaf scorch disease in real-time under two different lighting conditions (natural and artificial) for image acquisition. All models (SqueezeNet, AlexNet, VGG-16, and EfficientNet-B3) performed better under controlled lightening circumstances, and model performance decreased during natural sunlight situations. Several researchers also reported similar results that controlled lightening arrangements can avoid false recognition, decrease noise in the image acquisition zone and reduce the effect of fluctuating natural illumination circumstances in the field for better performance [61,62]. In the field experiment with controlled lightening arrangements, EfficientNet-B3 achieved the highest classification accuracy with 0.80 and 0.86 for initial and severe disease stages, respectively. The second-best model was VGG-16, which achieved the highest classification accuracy with 0.77 and 0.84 for initial and severe disease stages. AlexNet achieved slightly lower accuracy (0.72, 0.79) in comparison with VGGNet and EfficientNet-B3. Overall, EfficientNet-B3 achieved better results in all experiments than the other three (SqueezeNet, AlexNet, VGG-16) architectures, proving to be more suitable for disease classification in real-time.

## 5. Conclusions and Future Work

Deep learning approaches have recently become well-known for image data processing and target recognition in real-time applications. This paper focuses on identifying the initial and the severe stage of leaf scorch disease in strawberry plants with four trained CNNs models (SqueezeNet, EfficientNet-B3, VGG-16, AlexNet). The CNNs (SqueezeNet, EfficientNet-B3, VGG-16, AlexNet) models were trained at 80:20 images dataset ratios (for training and testing) and tested for the identification of leaf scorch disease infected plants.

The study validation results show that all the trained CNN models attained significant classification values of accuracy, F1-score, precision and recall. The performance accuracy of EfficientNet-B3 and VGG-16 was higher for the initial and severe stage of leaf scorch disease when compared with AlexNet and SqueezeNet. EfficientNet-B3 achieved 0.92, 0.97 classification accuracy for initial and severe stage leaf scorch disease, respectively. SqueezeNet recorded the lowest disease classification accuracy values in comparison with AlexNet, VGG-16 and EfficientNet-B3. It was also noticed that the severe stage of leaf scorch disease was more correctly classified than the initial stage of leaf scorch disease.

The field experiment results show that the EfficientNet-B3 and VGG-16 CNN model achieved significant disease classification performance with artificial lighting arrangements for better image acquisition in the strawberry field. Real-time field performance evaluation of CNN models results reported that the EfficientNet-B3 model achieved the highest values of recall (0.77, 0.85), precision (0.80, 0.87), and F-measure (0.78, 0.85) for the classification of the initial and severe stage of leaf scorch disease, respectively, during controlled lightening arrangements. However, there is a noticeable drop in CNN model performance in natural sunlight environments. The EfficientNet-B3 model performed best by achieving higher accuracy values in real-world field experiments, proving that more accurate deep learning systems can help better diagnose leaf scorch disease in strawberry crops. Furthermore, growers will quickly estimate the disease severity and take the essential precautions by applying appropriate pesticides. The proposed CNN models will make a significant contribution to better agriculture production.

The trained CNNs models will be used for targeted variable rate agrochemicals spraying systems in the strawberry crop for disease control. In future work, the number of image datasets will be increased so that the models can make more precise predictions in complex situations. We will also train CNN models on other leaf diseases. Additionally, CNN models will be trained for the identification of nutrient deficiency in strawberry plants.

## Figures and Tables

**Table 1:** Description of image dataset used for training, testing and validation.

Dataset Splitting Ratio	Training/Validation/Testing	Total Leaves Images	Healthy Leaves	Initial Disease Stage	Severe Disease Stage
80:20	Training	10,809	3595	3611	3603
	validation	1756	580	590	585
	testing	945	310	320	315

**Table 2:** CNN models validation results for the classification of disease infected strawberry leaves.

Model	Disease Stage	Precision	Sensitivity/ Recall	F1 Score	**Inference Time (ms)**
VGG-16	Initial	0.91	0.90	0.90	355
	Severe	0.96	0.95	0.95	349
AlexNet	Initial	0.88	0.89	0.88	109
	Severe	0.94	0.93	0.93	111
SqueezeNet	Initial	0.87	0.88	0.87	76
	Severe	0.93	0.92	0.92	66
EfficientNet-B3	Initial	0.92	0.91	0.91	212
	Severe	0.98	0.97	0.97	222

**Table 3:** CNNs models evaluation results in real-time for the classification of leaf scorch disease.

Model	Disease Stage	Precision	Sensitivity	F1 Score
VGG-16	Initial	0.70	0.67	0.68
	Severe	0.80	0.78	0.78
AlexNet	Initial	0.66	0.63	0.64
	Severe	0.75	0.73	0.73
SqueezeNet	Initial	0.64	0.61	0.62
	Severe	0.73	0.71	0.71
EfficientNet-B3	Initial	0.73	0.70	0.71
	Severe	0.83	0.81	0.81

**Table 4:** CNNs models evaluation results in real-time for the classification of leaf scorch disease in strawberry plants.

Model	Disease Stage	Precision	Sensitivity	F1 Score
VGG-16	Initial	0.78	0.75	0.76
	Severe	0.85	0.83	0.83
AlexNet	Initial	0.73	0.70	0.71
	Severe	0.80	0.78	0.78
SqueezeNet	Initial	0.71	0.68	0.68
	Severe	0.77	0.75	0.76
EfficientNet-B3	Initial	0.80	0.77	0.78
	Severe	0.87	0.85	0.85
